# Nutritional status, dietary habits, and their relation to cognitive functions: A cross-sectional study among the school aged (8–14 years) children of Bangladesh

**DOI:** 10.1371/journal.pone.0304363

**Published:** 2024-05-28

**Authors:** Mowshomi Mannan Liza, Simanta Roy, Mohammad Azmain Iktidar, Sreshtha Chowdhury, Azaz Bin Sharif

**Affiliations:** 1 Department of Public Health, North South University, Dhaka, Bangladesh; 2 Global Health Institute, North South University, Dhaka, Bangladesh; Bangabandhu Sheikh Mujib Medical University (BSMMU), BANGLADESH

## Abstract

**Background:**

Limited research addressed links between nutritional status, dietary habits, and cognitive functions in young children. This study assessed the status of cognitive functions and their association with nutritional status and dietary habits of school age children of Bangladesh.

**Methods:**

This cross-sectional multi-centre study was conducted on 776 participants in 11 conveniently selected educational institutions. A printed questionnaire with three sections (Section 1: background information, section 2: PedsQL™ Cognitive Functioning Scale, and section 3: semi-quantitative food-frequency questionnaire) was utilized for the data collection purpose. Sections 1 and 3 were self-reported by parents, and trained volunteers completed section 2 in-person along with the anthropometric measurements. Statistical analyses were done in Stata (v.16). Mean with standard deviation and frequencies with percentages were used to summarize quantitative and qualitative variables, respectively. Pearson’s chi-square test and Spearman’s rank correlation coefficient were used to explore bivariate relationships.

**Results:**

The mean age of the participants was 12.02±1.88 years, and the majority (67%) were females. The prevalence of poor cognitive function was 46.52%, and among them, 66.02% were females. In terms of body mass index (BMI), 22.44% possessed normal weight, 17.51% were overweight, and 5.19% were obese. This study found a statistically significant relationship between BMI and cognitive functions. Furthermore, different dietary components (e.g., protein, carbohydrate, fat, fiber, iron, magnesium) showed a significant (p<0.05 for all) weak positive correlation with cognitive function.

**Conclusion:**

BMI and dietary habits were associated with the cognitive function of young children in Bangladesh. Although the cross-sectional design of the study precludes causal relationships from being determined, the study finding deserves further examination via longitudinal research.

## Introduction

Child malnutrition is a critical global issue, accounting for approximately 11% of the global disease burden and over half of child deaths in developing countries [1]. More than 650 million people worldwide are overweight, and about 340 million children aged 5–19 years are overweight or obese [2]. The prevalence of childhood obesity has increased dramatically in metropolitan areas of Bangladesh, which was observed at less than 1% to 17.9% [3]. Also, micronutrient deficiencies are highly prevalent in children under five years, and it did not significantly improve over the past decade (2011–2021) [4].

The maturation and development of children’s central nervous systems and cognitive function are substantially influenced by nutrition [5]. Also, exposure to educational institution and social interactions play a crucial role in this regard. Therefore, nutritional stability is vital throughout the school years, which is a time of intense growth due to increased physical and mental demands [6,7]. Proximate principles and micronutrients are more important for children because of their increasing dietary needs [8]. According to WHO, the recommended daily calorie intake is 2100 kcal per person, which varies based on age and sex [9]. Fat, protein, vitamin A, thiamine, riboflavin, niacin, folic acid, vitamin B12, vitamin C, and iodine are essential for children’s growth and development, specifically, the calcium intake for children between the age of 10 to 14 is significant due to their rapid growth [9,10]. Micronutrient deficiencies, including iron, vitamin B12, folate, zinc, and vitamin D, are significantly associated with poor cognitive functions [11]. However, an estimated 25–27% of adolescents in Bangladesh are anaemic (Hb 12 g/dL), and 30% of 14–18-year-olds are iron-deficient (15%); as many as half of all school aged children (47–54%) do not have sufficient vitamin A [12]. Moreover, 60% of school children in Dhaka city aged 10 to 16 are not getting the recommended amount of protein, iron, and calcium [12]. Therefore, it is crucial to understand the effect of such deficiencies on the intellectual health of the children specifically on their cognitive functions.

Several prior studies tried to explain the effects of nutrition on the cognitive function of children. For example, a review article reported a correlation between obesity, cognitive and motor function declines, and altered brain plasticity [13]. In addition, nutritional deficiency can cause more absenteeism, earlier drop out, and poor performance in the classroom among the school aged children [14]. However, a prospective cohort study failed to determine any relationship between cognitive domain scores and nutritional status, although the primary focus of the study was patients with Alzheimer’s disease and mild cognitive impairment [15,16]. Another study conducted in South India, indicates that cognitive function throughout middle childhood are vital in association with early-life malnutrition and future health. They evaluated cognitive achievements in reading and mathematics by questions with varying difficulty level to better understand their impact on cognitive functions [17]. However, to date there is no study conducted in Bangladesh to explore the relationship between nutrition and cognitive function, specifically among school aged children. Therefore, [17,18] the aim of this study was to investigate the relationship between the nutritional status, dietary habits, and cognitive functions among school aged children in Bangladesh.

## Methods

### Study design, setting, and sample

**Fig 1:**
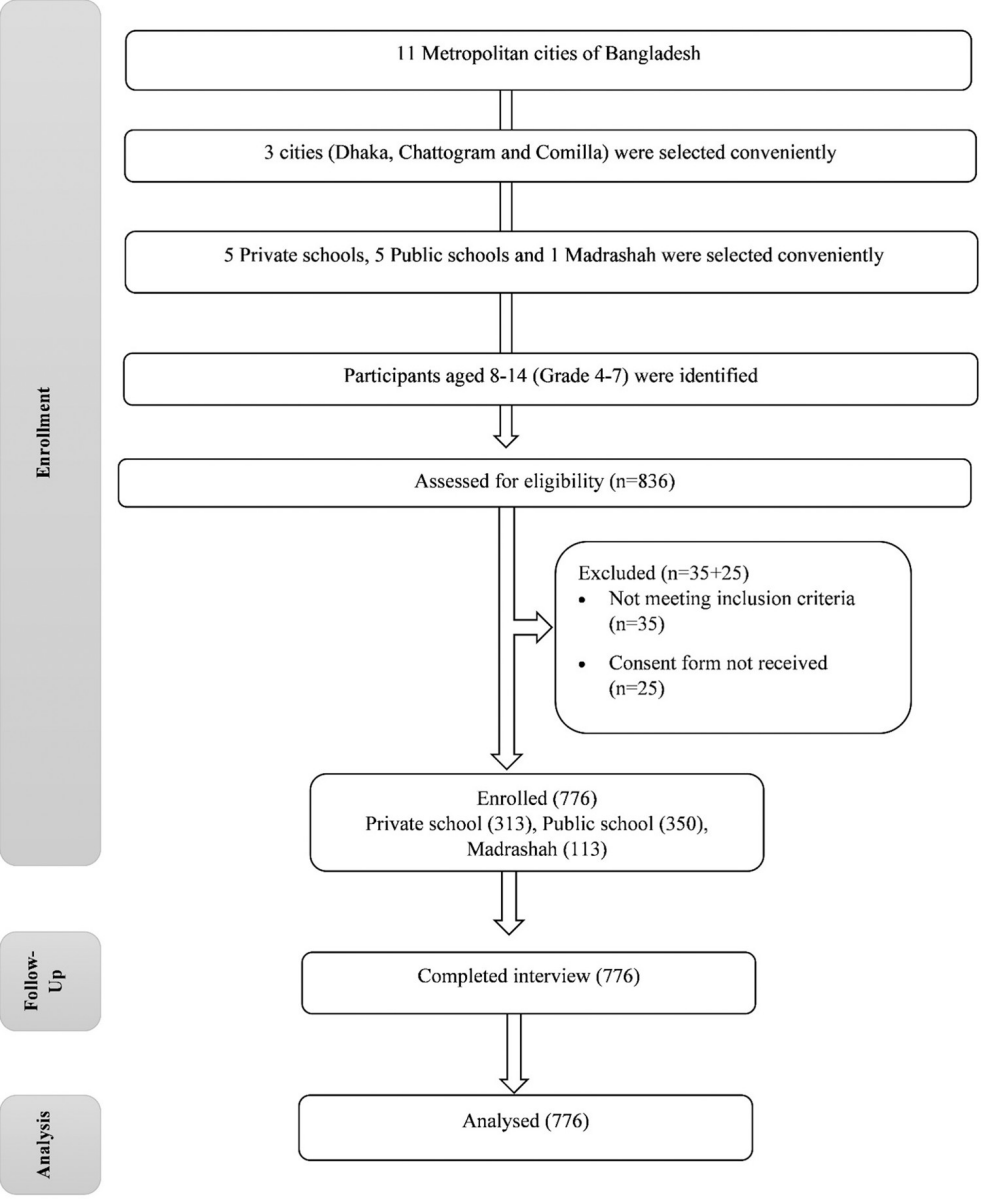
CONSORT flow diagram.

Children between the ages of 8 to 14, who were enrolled in grades 4 to 7 at five private schools, five public schools, and one Madrasah (a facility specifically designed for Islamic education and culture) in Bangladesh, were included in this cross-sectional study. Convenient sampling was used to select the institutions from three metropolitan cities of Bangladesh (Dhaka, Chattogram, and Cumilla) **(Fig 1)**. All students from these institutions were considered for participation upon fulfilling the inclusion criteria. The study excluded participants with pre-existing cognitive decline, mental health conditions, or any other illness that can affect cognitive functions. Eligible participants underwent a face-to-face interview by trained volunteers, while the parent responses were collected using a self-reported questionnaire.

Informational brochures, parental permission forms, questionnaires, and instructions were distributed to every participant in the selected schools. The brochures included contact details of a study volunteer in case any question arises during the questionnaire completion process. Out of 836 eligible students, 776 children (response rate 92.8%) who submitted written parental consent and questionnaire within a week were considered for cognitive function assessment interviews and anthropometric measurements. Data were acquired from May to November of 2021 with approval from the Institutional Review Board of North South University (Approval no-2022/0R-NSU/IRB/1005). The study was carried out in compliance with the Helsinki Declaration 1964.

### Pretesting

Twenty participants from government and private schools each participated in pre-test to determine the feasibility and validity of this research. To accelerate data gathering without sacrificing data quality, necessary changes were made. On the recommendations of the pilot participants, the inclusion of the helpline number in brochures was taken into consideration.

### Measures

This study employed a semi-structured questionnaire with three parts. Part 1 included questions about age, gender, residence, family type, family income, parental education status, birth order and delivery method during birth, EPI vaccination status, duration of breastfeeding, and deworming status. Sections 2 and 3 contained the PedsQL^TM^ Cognitive Functioning Scale [19,20], and a 39-item semi-quantitative food-frequency questionnaire (FFQ) [21], respectively. Both of these questionnaires were pre-validated and were used to evaluate cognitive functions and dietary intakes, respectively. Sections 1 and 3 were sent to the parents with clear instructions on how to complete them. A face-to-face interview of participants was conducted after they had returned with a completed questionnaire with parental consent. Trained volunteers completed the remaining portions of the questionnaire (section 2: PedsQL^TM^ Cognitive Functioning Scale) with information directly obtained from the child via face-to-face interview. Additionally, anthropometric measurements were also taken during the interview.

### PedsQL^TM^ cognitive functioning scale

The PedsQL^TM^ Cognitive Functioning Scale consists of six questions. This scale was developed through focus group discussions, cognitive interviews, pre-testing, and field-testing measurement development techniques which was used in one of our prior studies [22,23]. A five-point likert scale was used to assess this scale, with 0 denoting never, 1 denoting nearly never, 2 denoting sometimes, 3 denoting often, and 4 denoting almost always. All responses were reverse-scored and then linearly translated to a 0–100 scale (0 = 100, 1 = 75, 2 = 50, 3 = 25, 4 = 0), in accordance with established scoring protocols. Any score below the mean was considered as poor cognitive functioning and higher scores indicated higher functioning.

### Nutritional status

During the data collecting process, trained data collectors took standardized anthropometric measures such as height, and weight. A commercial, non-elastic measuring tape was used to take height measurements. The participants were asked to stand with their heads held high, legs dangling loosely, and barefooted. The highest point of the head was indicated on large white cardboard affixed to the wall. The length from the floor to that point was calculated using tape. The height was rounded to the nearest 0.1 cm. Body weight was measured using an electronic weight scale in whole numbers with and without shoes, coats, or other bulky accouterments. The measures were then transformed using WHO AnthroPlus software for assessing growth data for the age category of 5–19 years into BMI-for-age-Z-score (BAZ). Hence, BMI was calculated and categorized accordingly.

### Dietary status

A pre-validated questionnaire including the common food items was used to record the participant’s dietary intake using a 7-day recall food diary [24]. Guardians were asked about the number of months per year they consumed each item, the number of days per week they ate the thing during those months, the number of times in a typical day they ate the item, and the amount they consumed on average each time. Pictures of different locally used plates and utensils were used to define the portion sizes. The nutritional value of the food items was calculated using the food composition table for Bangladesh [25].

### Statistical analysis

All analyses were performed using STATA MP Version 16. Descriptive statistics, such as mean, standard deviations, frequencies, and percentages were calculated. Association between two categorical variables was performed using the chi-squared test. The correlation between cognitive function and dietary nutrient intake was evaluated using Spearman’s correlation coefficient. A p-value of <0.05 was considered statistically significant.

## Result

Background information on the study participants is presented in **Table 1**. The mean (±SD) age of the school-aged children between 8 and 14 years was 12.02 (±1.80) years. Among the 776 participants, the majority were female (67.31%), and lives in urban areas (61.48%). About 78% of the participants belong to a nuclear family, and most of them have birth orders first or second. Most of the participants’ family income was 27,546.21 BDT or more. Around half of the responding guardians had less than 10 years of schooling experience. Almost 68% of the children were born to mothers via normal vaginal delivery (NVD). The rate of EPI vaccination was high (90.56%) among the respondents, and most of them were breastfed (73.28%) for more than a year. Majority of the participants either regularly (48.1%) or occasionally (49.08%) dewormed.

**Table 1:** Background information of study participants (n = 776).

Variables	Frequency	Percentage
**Age (in years), mean±SD**	12.02±1.80	
**Gender**		
Male	254	32.69
Female	522	67.31
**Residence**		
Rural	299	38.52
Urban	477	61.48
**Type of family**		
Nuclear	602	77.59
Joint	174	22.41
**Birth Order**		
1^st^ or second	574	74.01
Third or more than third	202	25.99
**Monthly family income (in BDT) mean±SD**	27546.21**±** 66198.76	
**Guardians’ education**		
Less or Equal to 10 years	407	52.5
More than 10 years	369	47.5
**Mode of delivery**		
Do not know	45	5.86
NVD by others	185	23.8
NVD by Doctor	342	44.02
Caesarean section	204	26.32
**EPI vaccination**		
No	73	9.44
Yes	703	90.56
**Duration of breastfeeding (in months)**		
< 6 months	84	10.78
6–12 months	124	15.93
12–24 months	198	25.49
>24 months	371	47.79
**Deworming**		
Never	22	2.82
Occasionally	381	49.08
Regularly (3 monthly)	373	48.1

SD, standard deviation; BDT, Bangladeshi taka, NVD, Normal Vaginal Delivery.

**Table 2** demonstrates the nutritional status of the study participants. In the BMI category, 22.44% were underweight, 17.51% were overweight, and 5.19% were obese.

**Table 2:** Nutritional status of study participants.

Variables	Frequency	Percentage
**BMI (Body Mass Index) (n = 771)**		
Underweight (<-2SD)	173	22.44
Normal weight (< +1SD to < -2SD)	423	54.86
Overweight (>+1SD)	135	17.51
Obese (>+2SD)	40	5.19

Average daily intakes of nutrients with their reference values from 7-day recall food diary are presented in **Table 3**. The table showed different nutrient deficiencies (e.g., fibre, iron, calcicum) to varied extend.

**Table 3:** Average daily intake of dietary nutrients with their reference value 7-day recall food diary.

Nutrients	*Reference Value*	*Mean±SD*	Nutrients	*Reference Value*	*Mean±SD*
Energy (Kcal)	35 to 65 kcal/kg[26]	913.38±310.24	Vitamin A (μg)	600 [27]	606.43±391.35
Protein (g)	0.8g/kg[28]	47.47±25.99	Vitamin E (mg)	11 [27]	2.65±1.71
Carbohydrate (g)	191.7 ± 58.9[29]	81.75±52.75	Vitamin D (μg)	15 [27]	1.33±0.86
Fat (g)	-	12.56±7.30	Vitamin C (mg)	45 [27]	40.76±26.30
Dietary Fibre (g)	25-31g[30]	9.45±6.10	Thiamine (mg)	0.9 [27]	0.50±0.31
Iron (mg)	8[27]	6.95±4.18	Riboflavin (mg)	0.9 [27]	0.72±0.45
Calcium (mg)	1300 [27]	509.66±267.94	Niacin (mg)	12 [27]	15.91±8.63
Magnesium (mg)	240 [27]	139.04±85.94	Pyridoxine (mg)	1 [27]	0.77±0.46
Phosphorus (mg)	1250 [27]	614.74±346.02	Folate (μg)	600 [27]	110.15±71.09
Sodium (mg)	1200 [27]	352.18±198.05			
Potassium (mg)	2500 [27]	1139.58±650.72			
Zinc (mg)	8 [27]	7.39±4.42			
Copper (mg)	0.7 [27]	1.13±0.63			

**Table 4** shows that 46.5% of participants had a poor cognitive function, and the remaining 53.48% had a good cognitive function. Among them, females with poor cognitive function, 66.02%, and their mean ages were 12.02±1.88 and 12.0±1.66. Most of the participants were from urban areas with poor and good cognitive function 61.5% and 55.9%, respectively, and in the case of nuclear families’ participants with poor cognitive function were 78.65%. Roughly 74% had first and second birth orders with poor cognitive function. Around half of the responding guardians had poor cognitive functional children. For most of the participants, around 70% had poor cognitive function; the mode of delivery was NVD. Around 91% of the participants were EPI-vaccinated with poor cognitive function. Almost half (47.59%) of the participants with poor cognitive function were breastfed for more than 24 months. Participants with poor cognitive function had 52.68% dewormed occasionally. Children with poor cognitive function had a body mass index (BMI) distribution as 23.5% underweight, 21.08% overweight, and the reamining 3.31% obese.

**Table 4:** Cognitive function of the study participants (n = 776).

Variables	Poor Cognitive Function (n = 361, 46.52%)	Good Cognitive Function (n = 415, 53.48%)	p-value
	Frequency (%)	Frequency (%)	
**Age (in years), mean±SD**	12.02±1.88	12.05±1.66	0.61^ψ^
**Gender**			
Male	123 (33.98)	118(28.47)	0.09^€^
Female	238 (66.02)	297(71.53)	
**Residence **			
Rural	139 (38.5)	183(44.1)	0.12^€^
Urban	222 (61.5)	232(55.9)	
**Type of family **			
Nuclear	284 (78.65)	321(77.34)	0.66^€^
Joint	77 (21.35)	94(22.66)	
**Birth Order**			
1^st^ or second	265 (73.82)	293 (73.62)	0.95^€^
Third or more than third	94 (26.18)	105 (26.38)	
**Monthly family income (in BDT) mean±SD**	27546.21**±** 66198.76		0.50 ^ψ^
**Guardians’ education**			
Less or Equal to 10 years	187 (51.9)	234(56.34)	0.31^€^
More than 10 years	174 (48.1)	181(43.66)	
**Mode of delivery **			
Do not know	16 (4.43)	26(6.27)	0.58^€^
NVD by others	94 (26.04)	99(23.86)	
NVD by Doctor	157 (43.49)	189(45.54)	
C/S	94 (26.04)	101(24.34)	
**EPI vaccination**			
No	31 (8.59)	43(10.36)	0.4^€^
Yes	330 (91.41)	372(89.64)	
**Duration of breastfeeding **			
< 6 months	43 (11.9)	41 (9.98)	0.77^€^
6–12 months	55 (15.3)	72 (17.27)	
12–24 months	91 (25.21)	102 (24.57)	
>24 months	172 (47.59)	200 (48.18)	
**Deworming**			
Never	9 (2.54)	14 (3.46)	0.13^€^
Occasionally	190 (52.68)	189(45.43)	
Regularly (3 monthly)	162 (44.79)	212(51.11)	
**BMI (body mass index) Category **			
Underweight	78 (23.5)	83 (51.55)	***0*.*048*** ^€^
Normal weight	173 (52.11)	228(58.31)	
Overweight	70 (21.08)	55(14.07)	
Obese	11 (3.31)	25(6.39)	

SD, standard deviation; BDT, Bangladeshi taka; NVD, Normal Vaginal Delivery

Ψ, Spearman correlation p-value

€, Chi-square test p-value.

There was a significant but weak positive correlation between Cognitive function and the intake of protein, carbohydrate, fat, B vitamins, some water-soluble (Thiamine, Riboflavin, Niacin, Pyridoxine, Folate), fat-soluble vitamins (Vitamin A, D, E), and minerals including iron, magnesium, Phosphorus, Sodium, Potassium, zinc, Copper (p < 0.05) (**Table 5**).

**Table 5:** Spearman’s correlation between Cognitive function and daily nutrients intake.

Nutrients	*r* *	*p*-value**	Nutrients	*r* *	*p*-value**
Energy (Kcal)	0.006	**0.86**	Vitamin A (μg)	0.1	**0.02**
Protein (g)	0.1	**0.02**	Vitamin E (mg)	0.1	**0.02**
Carbohydrate (g)	0.1	**0.02**	Vitamin D (μg)	0.1	**0.02**
Fat (g)	0.1	**0.03**	Vitamin C (mg)	0.1	**0.02**
Dietary Fibre (g)	0.1	**0.02**	Thiamine (mg)	0.1	**0.03**
Iron (mg)	0.1	**0.03**	Riboflavin (mg)	0.1	**0.03**
Calcium (mg)	0.03	**0.37**	Niacin (mg)	0.1	**0.02**
Magnesium (mg)	0.1	**0.03**	Pyridoxine (mg)	0.1	**0.03**
Phosphorus (mg)	0.1	**0.03**	Folate (μg)	0.1	**0.02**
Sodium (mg)	0.1	**0.03**			
Potassium (mg)	0.1	**0.03**			
Zinc (mg)	0.1	**0.03**			
Copper (mg)	0.1	**0.02**			

* r is Spearman’s correlation coefficient.

** p-value is for Spearman’s correlation test, p < 0.05 is statistically significant.

## Discussion

The study’s results emphasize the significant correlation between BMI and cognitive function in school aged children. Although it is difficult to isolate the specific impact of nutrition due to its intricate interaction with factors such as demography, socioeconomic position, health, and genetic influences, this study supports previous research that emphasizes the crucial role of nutrition in cognitive development [16,31,32]. This study also corroborates global patterns of childhood obesity and overweight, consistent with findings from Dhaka city, which indicate the increased prevalence of overweight and obesity among school-going children [33]. The results of this study indicate that 46.52% of the participants showed poor cognitive function, which is consistent with a study conducted in Bangladesh. The finding highlights the importance of raising parental awareness regarding the cognitive health of children in Bangladesh.

The results of this study found that among school aged children, in terms of BMI, 3.31% obese and 21.08% overweight children had poor cognitive functions. Similarly, a study on children aged between 9–10 years old also reported a potential link between BMI and cognitive function [34]. Another study on young medical students emphasized a correlation between BMI and cognitive function [35]. This study also provides support that BMI is associated with cognitive functions. The possible mechanism can include changes in cortical volumes and other morphological alterations, especially in regions of the prefrontal cortex responsible for executive function [36]. However, a better understanding of this issue can be achieved through a nationwide study on a diverse population with the help of clinical examination of cognitive function.

Several prior reviews highlighted the role of different nutrients in the development and proper functioning of brain [10,36]. For a better cognitive function, it is necessary to consume right amounts of protein and energy, in addition to the essential micronutrients [37]. Using a sizable sample, this study found deficiencies in a number of nutrients and different dietary nutrients (e.g., protein, carbohydrate, fat, iron, calcium) also showed a very weak positive significant correlation with cognitive function. The self-reported data on dietary intake and non-clinical methods of cognitive function assessment can be a reason for this weak relationship. Still, this finding can be a representation of the bigger picture which can be assessed in a larger study [37,38]. Therefore, further study is required to investigate the consequences of dietary consumption on cognitive function more precisely.

To our best knowledge, this is the first study ever conducted in Bangladesh to investigate the association between cognitive function, nutritional status, and dietary intake among school-going children. The questionnaires used in this research are more reliable since they were adapted from previously validated scales. The large sample size also helps ensure the reliability and validity of the results. However, this study has some limitations. First, the cross-sectional design and unavailability of multivariate analysis preclude causal relationships from being determined. However, it deserves further exploration through longitudinal studies. Larger studies and clinical trials examining the effect of different nutrients on the cognitive function of young children are warranted. Second, the measurement of cognitive function may not be accurate, considering the absence of clinical tests. Third, there is a higher risk of recall biases in terms of FFQ. Also, the convenience sampling techniques may cause selection bias and limit the generalizability of the study results.

## Conclusion and recommendation

According to the results of this research, this study found an association between BMI and cognitive function and identified several nutrients weakly associated with the cognitive function of school aged children. However, we could not draw any direct association between cognitive function, dietary habits, and nutritional status. Further longitudinal studies are required to draw a better conclusion.

## Supporting information

S1 Checklist STROBE statement—Checklist of items that should be included in reports of observational studies. (DOCX)

S1 Dataset (XLSX)

## References

[1] Emmanuel K. Assessment of the Nutritional Status of Junior High School Students–Evidence from Mfantseman Municipality of Ghana. Science Journal of Public Health. 2013;1: 222. doi: 10.11648/j.sjph.20130105.16

[2] Meo SA, Altuwaym AA, Alfallaj RM, Alduraibi KA, Alhamoudi AM, Alghamdi SM, et al. Effect of obesity on cognitive function among school adolescents: A cross-sectional study. Obes Facts. 2019;12: 150–156. doi: 10.1159/000499386 30865949 PMC6547262

[3] Rahman S, Islam MT, Alam DS. Obesity and overweight in Bangladeshi children and adolescents: A scoping review. BMC Public Health. 2014;14: 1–8. doi: 10.1186/1471-2458-14-70 24450958 PMC3912929

[4] icddr,b—Press Releases. [cited 8 Jan 2024]. Available: https://www.icddrb.org/quick-links/press-releases?id=139&task=view.

[5] Handa R, Prasad R. Effect of undernutrition on cognitive development of children. 2010. doi: 10.47556/J.IJFNPH.3.2.2010.3

[6] Taras H. Nutrition and student performance at school. Journal of School Health. 2005;75: 199–213. doi: 10.1111/j.1746-1561.2005.00025.x 16014126

[7] Reber M. Development during Middle Childhood: The Years from Six to Twelve. Journal of Developmental & Behavioral Pediatrics. 1986. doi: 10.1097/00004703-198604000-00017

[8] Isaacs E, Oates J. SUPPLEMENT Nutrition and cognition: assessing cognitive abilities in children and young people. 2008; 4–24. doi: 10.1007/s00394-008-3002-y 18683026

[9] Woodruff BA, Duffield A. Anthropometric assessment of nutritional status in adolescent populations in humanitarian emergencies. Eur J Clin Nutr. 2002;56: 1108–1118. doi: 10.1038/sj.ejcn.1601456 12428177

[10] Ekstrand B, Scheers N, Rasmussen MK, Young JF, Ross AB, Landberg R. Brain foods—the role of diet in brain performance and health. Nutr Rev. 2021;79: 693–708. doi: 10.1093/nutrit/nuaa091 32989449

[11] Singh S, Awasthi S, Kumar D, Sarraf SR, Pandey AK, Agarwal GG, et al. Micronutrients and cognitive functions among urban school-going children and adolescents: A cross-sectional multicentric study from India. PLoS One. 2023;18: e0281247. doi: 10.1371/journal.pone.0281247 36730336 PMC9894395

[12] Alam N, Roy SK, Ahmed T, Ahmed AMS, Alam N, Kumar S, et al. Linked references are available on JSTOR for this article: Nutritional Status, Dietary Intake, and Relevant Knowledge of Adolescent Girls in Rural Bangladesh. 2016;28: 86–94. 10.3329/jhpn.v28i1.4527 PMC2975850 20214090

[13] Wang C, Chan JSY, Ren L, Yan JH. Obesity Reduces Cognitive and Motor Functions across the Lifespan. Neural Plast. 2016;2016. doi: 10.1155/2016/2473081 26881095 PMC4737453

[14] Prithviraj Karak*, Rajkumar Maiti PD and AK. ASSESSMENT OF NUTRITIONAL STATUS OF SCHOOL CHILDREN IN RURAL AND URBAN AREAS OF BANKURA, WEST BENGAL. IJPSR. 2018;Vol. 9: 338–345.

[15] Doorduijn AS, Visser M, van de Rest O, Kester MI, de Leeuw FA, Boesveldt S, et al. Associations of AD biomarkers and cognitive performance with nutritional status: The NUDAD project. Nutrients. 2019;11. doi: 10.3390/nu11051161 31126170 PMC6566264

[16] Stein NDF and AD. Risk factors affecting child cognitive development: A summary of nutrition, environment, and maternal-child interaction indicators for sub-Saharan Africa. HHS Public Access. 10.1017/S2040174415001427 PMC4800975 26358240

[17] Acharya Y, Luke N, Haro MF, Rose W, Russell PSS, Oommen AM, et al. Nutritional status, cognitive achievement, and educational attainment of children aged 8–11 in rural South India. PLoS One. 2019;14: e0223001. doi: 10.1371/journal.pone.0223001 31596845 PMC6784908

[18] Huq AKO, Mony SK, Chowdhury TD, Uddin I, Rahim ANMB, Jahan T, et al. Nutritional status and dietary patterns of children with attention deficit hyperactivity disorder in Bangladesh. Int J Publ Health Sci. 2023;12: 1102–1111. doi: 10.11591/IJPHS.V12I3.22553

[19] Liza MM, Iktidar MA, Roy S, Jallow M, Chowdhury S, Tabassum MN, et al. Gadget addiction among school-going children and its association to cognitive function: a cross-sectional survey from Bangladesh. BMJ Paediatr Open. 2023;7: e001759. doi: 10.1136/bmjpo-2022-001759 36808098 PMC9944298

[20] Varni JW, Sherman SA, Burwinkle TM, Dickinson PE, Dixon P. The PedsQLTM Family Impact Module: Preliminary reliability and validity. Health Qual Life Outcomes. 2004;2: 1–6. doi: 10.1186/1477-7525-2-55/TABLES/3 15450120 PMC521692

[21] Chen Y, Ahsan H, Parvez F, Howe GR. Validity of a food-frequency questionnaire for a large prospective cohort study in Bangladesh. Br J Nutr. 2004;92: 851–859. doi: 10.1079/bjn20041277 15533275

[22] Liza MM, Iktidar MA, Roy S, Jallow M, Chowdhury S, Tabassum MN, et al. Gadget addiction among school-going children and its association to cognitive function: A cross-sectional survey from Bangladesh. BMJ Paediatr Open. 2023;7: 1–7. doi: 10.1136/bmjpo-2022-001759 36808098 PMC9944298

[23] Varni JW, Sherman SA, Burwinkle TM, Dickinson PE, Dixon P. The PedsQLTM Family Impact Module: Preliminary reliability and validity. Health Qual Life Outcomes. 2004;2: 1–6. doi: 10.1186/1477-7525-2-55 15450120 PMC521692

[24] Chen Y, Ahsan H, Parvez F, Howe GR. Validity of a food-frequency questionnaire for a large prospective cohort study in Bangladesh. British Journal of Nutrition. 2004;92: 851–859. doi: 10.1079/bjn20041277 15533275

[25] Shaheen N. Food composition table for Bangladesh. Igarss 2014. 2014.

[26] Davidson’s Principles and Practice of Medicine: Davidson’s Principles and…—Google Books. [cited 24 Jan 2024]. Available: https://books.google.com/books/about/Davidson_s_Principles_and_Practice_of_Me.html?id=vhl2EAAAQBAJ.

[27] Children | Linus Pauling Institute | Oregon State University. [cited 24 Jan 2024]. Available: https://lpi.oregonstate.edu/mic/life-stages/children.

[28] Faizan U, Rouster AS. Nutrition and Hydration Requirements In Children and Adults. StatPearls. 2023 [cited 24 Jan 2024]. Available: https://www.ncbi.nlm.nih.gov/books/NBK562207/.

[29] Nguyen AN, Santos S, Braun KVE, Voortman T. Carbohydrate Intake in Early Childhood and Body Composition and Metabolic Health: Results from the Generation R Study. Nutrients. 2020;12: 1–11. doi: 10.3390/nu12071940 32629760 PMC7399886

[30] Kranz S, Brauchla M, Slavin JL, Miller KB. What Do We Know about Dietary Fiber Intake in Children and Health? The Effects of Fiber Intake on Constipation, Obesity, and Diabetes in Children. Advances in Nutrition. 2012;3: 47. doi: 10.3945/an.111.001362 22332100 PMC3262613

[31] Honja Kabero T, Bosha T, Feleke FW, Haile Weldegebreal D, Stoecker B. Nutritional Status and Its Association with Cognitive Function among School Aged Children at Soddo Town and Soddo Zuriya District, Southern Ethiopia: Institution Based Comparative Study. Glob Pediatr Health. 2021;8. doi: 10.1177/2333794X211028198 34263015 PMC8246583

[32] Rosales FJ, Reznick JS, Zeisel SH. Understanding the role of nutrition in the brain and behavioral development of toddlers and preschool children: Identifying and addressing methodological barriers. Nutr Neurosci. 2009;12: 190–202. doi: 10.1179/147683009X423454 19761650 PMC2776771

[33] Hassan NE, El-Masry SA, Fouad WA, Sherif L, Elwakkad A, Anwar M, et al. Prevalence of metabolic syndrome among obese school students. e-SPEN. 2011;6. doi: 10.1016/j.eclnm.2011.09.005

[34] Dennis E, Manza P, Volkow ND. Socioeconomic status, BMI, and brain development in children. Transl Psychiatry. 2022;12: 1–10. doi: 10.1038/s41398-022-01779-3 35075111 PMC8786961

[35] Khade Y, Kumar AVS, Maruthy KN, Sasikala P. Does body mass index influence cognitive functions among young medical students? Clin Epidemiol Glob Health. 2021;12: 100874. doi: 10.1016/j.cegh.2021.100874

[36] Dennis E, Manza P, Volkow ND. Socioeconomic status, BMI, and brain development in children. Translational Psychiatry 2022 12:1. 2022;12: 1–10. doi: 10.1038/s41398-022-01779-3 35075111 PMC8786961

[37] Bryan J, Osendarp S, Hughes D, Calvaresi E, Baghurst K, Van Klinken JW. Nutrients for cognitive development in school-aged children. Nutr Rev. 2004;62: 295–306. doi: 10.1111/j.1753-4887.2004.tb00055.x 15478684

[38] Kim JY, Kang SW. Relationships between Dietary Intake and Cognitive Function in Healthy Korean Children and Adolescents. J Lifestyle Med. 2017;7: 10–17. doi: 10.15280/jlm.2017.7.1.10 28261556 PMC5332116

