# Supplementary material for: Nutritional status, dietary habits, and their relation to cognitive functions: A cross-sectional study among the school aged (8–14 years) children of Bangladesh
Source: PLoS One. 2024 May 28;19(5):e0304363. doi: 10.1371/journal.pone.0304363 (PMC11132469; doi:10.1371/journal.pone.0304363)
Supplement: Attachment — Submitted filename: Reviewer Comments_PONE-D-23-15685.docx [file pone.0304363.s003.docx]

**Reviewer Comments**

This is an interesting study on the association between nutritional status, dietary habits and cognitive function among children in Bangladesh. Nutritional status and dietary habits is crucial for cognitive development of children.

I propose to address the following issues.

Abstract:

Line 16: May the research is not limited worldwide. The author needs to focus on Bangladesh and need to give background about Bangladesh.

Introduction:

Line 49: The author stressed on the need of only micronutrients, however, macronutrients like protein and fat is also important for the development of nervous system.

Line 72: To develop proper rationale of the study, author need to provide some background or previous studies in the related field done in Bangladesh (if have).

Methods:

Line 81: Why did you choose children between 8-12 years, however, in the title it has been mentioned that the study was done in school going children. Age range of school going children could 5 to 15 Years. As the age range 8-14 years, is this representative to the schooling children?

Line 106: Is the PedsQL and FFQ validated in Bangladesh? How the author ensured the quality of FFQ as these needs to depends on the memory of children for their food intake.

Line 119: The does not need to include questions in this section. The author can upload questions file separately as supplementary file.

Line 140: From the existing anthropometric data, it was possible to calculate weight-for height Z score, height for age z score to categorize wasting and stunting.

Line 150: The author conducted few simple statistical analyses like regression and descriptive analysis. It might be better to perform few advanced statistical analyses like logistic regression.

Results:

Line 176: In Table 2 author need to provide the BMI for age Z score cut-off points for BMI categories.

Discussion:

It is an existing knowledge that poor nutrition and dietary intake interfere with cognitive development in children. Though the study is the first ever study (according to author declaration), it was better to draw some novel inference from the findings and discuss accordingly.
