# Supplementary material for: Nutritional status, dietary habits, and their relation to cognitive functions: A cross-sectional study among the school aged (8–14 years) children of Bangladesh
Source: PLoS One. 2024 May 28;19(5):e0304363. doi: 10.1371/journal.pone.0304363 (PMC11132469; doi:10.1371/journal.pone.0304363)
Supplement: Attachment — Submitted filename: point_by_point_response.docx [file pone.0304363.s004.docx]

**Response to Reviewer Comments**

**Reviewer #1:** This cross-sectional study focused on dietary habits, nutritional status and cognition among children in Bangladesh. Relations/relationships are generally identified through regression. As there were 776 children included within the study unsure why only correlations were performed. There were areas that needed to be rewritten for grammar and syntax. The cognitive functioning scale appears more for ADHD or other behavioral disorders as opposed to cognitive status based on taking a test as is done with other assessments. As no other measurements were assessed (e.g. status in school or test scores), it is challenging to state cognitive status was poor or good. Even though stunting and weight are considered ways to measure nutrition, they are not the only ones and are more for anthropometrics. Curious if skinfolds or other techniques were used to confirm nutritional status.

*Thank you for the review. The manuscript has been updated with a thorough grammar check. The cognitive assessment tool has been validated for use in Bangladesh. However, an assessment through a physician is definitely the best method to assess cognitive function, which could not be done considering the resource limitation. Also, academic performance was not considered due to the wide variability in assessments across institutions, which might lead to a biased evaluation. For example, good performance in high-ranking and low-ranking schools cannot be evaluated on a similar scale. We mentioned a point-by-point response to all the comments below.*

**Title:** School-going may need to be revised to possibly School Children or Elementary or specific age groups of the children who were included in this study.

***Our response:*** *The age is now included in the title. This research only included participants who are currently attending school.*

**Abstract:** in the methods mentioned qualitative information, none were included within the results. Curious if qualitative variables were used in a different context than actual interview or text-based data obtained through the interviews, please clarify. For the results, indicate if the association between BMI and cognitive function were positive or negative. Clarify which or provide examples of those nutrients that were associated with cognitive function.

***Our response:*** *This is purely a quantitative study, and no qualitative information was gathered. In the method section, we stated qualitative variables, not qualitative information.*

*The questionnaire was mostly comprised of three sections. Section 1 included questions about age, gender, residence, family type, family income, parental education status, birth order and delivery method during birth, EPI vaccination status, duration of breastfeeding, and deworming status. Sections 2 and 3 contained the PedsQL Cognitive Functioning Scale and a 39-item semi-quantitative food-frequency questionnaire (FFQ), respectively. All data mentioned in the method has been mentioned in tables.*

*A summary of the result has been provided in the description. The association between BMI and cognitive function was assessed using a chi-square test; therefore, the direction of the relationship was not mentioned. Examples of different nutrients that were associated with cognitive function were mentioned in tables 3 and , rescpectively.*

**Introduction:**
In the first paragraph, expand if it is all during elementary years or only during early childhood, etc. The first three paragraphs of the introduction can be condensed and synthesized for clarity as essentially the information from these paragraphs are the same.
Cognitive functions are different dependent on the growth of the child, so young children <5 years will have different functions that a child who is 6-12 years old and even different between that age group. Please expand on the age that is the focus and what other studies in developing countries have shown this relationship between cognition and nutrition and why this study is important for those in Bangladesh. Please be sure to write the research question as open ended as opposed to yes/no

***Our response:*** *The manuscript has been modified according to the suggestion. Regarding the age of the participants, the study was focused on school-going children in their elementary years. Due to wide variations across schools (e.g., age of starting school, inclusion of pre-school), the stated age group in this study was considered to better represent this population.*

**Methods:**
For the pretesting, expand on who these participants consisted of – children or other age groups. Also, indicate how 20 was appropriate for this pretesting (e.g. reference).

***Our response:*** *We included 20 samples from both Government (20) and Private schools (20) for pre-testing. So, the total number is 40, which is standard (1). However, the line has been modified to make it more precise.*

Line 135: it should be bare foot instead of ‘naked feet’

***Our response:*** *It has been updated according to the reviewer’s suggestion.*

Line 137: indicate if body weight was taken to the tenth or whole number.

***Our response:*** *It has been updated in the manuscript that the body weight was measured in whole numbers.*

For the statistical analysis indicate the statistical significance.

***Our response:*** *It has been updated that a p-value <0.05 was considered for statistical significance.*

For the public involvement, unsure the purpose of this portion as mentioned in the pretesting how this was achieved. How were parents involved in the study design and why? Please clarify this portion.

***Our response:*** *This information is now detailed in the manuscript for better clarity. The respective section now reads:*

*Members of the public were involved in several stages of the study, including design and conduct. We received input from children and their parents during the pilot and implemented them in our study design. Also, informational brochures, parental permission forms, questionnaires, and instructions were distributed to every participant in the selected schools. The brochures included contact details of a study volunteer in case any question arises during the questionnaire completion process. We received feedback and queries from the parents, which ensured quality data.*

**Results:**
For table 2, thin is not the classification based on BMI, it would be underweight.

***Our response:*** *this correction has been made.*

For table 4, please place the actual p-values and not just <0.05.

***Our response:*** *The actual p-value is now reported as per your suggestion.*

For dietary intake, it would be relevant to include the actual nutrients to determine if these children were consuming less than the requirements as opposed to just including the correlation values.

***Our response:*** *In Table 3, we included the actual nutrient values along with the reference value.*

**Discussion:**
In the first sentence, mentions that BMI was statistically significant with cognitive function, yet the p-value was 0.05, so it appears there was no significance as p<0.05 appeared to be the mechanism to detect significance.

***Our response:*** *The p-value for the relationship between BMI and cognitive function was 0.048, which was rounded to 0.05 considering two decimal points. However, we updated it for better understanding.*

Much of the discussion included results, which can be eliminated and instead focus on why these results were similar or not to other studies.

***Our response:*** *Thanks for the suggestion. The discussion has been modified accordingly.*

For references that were included, may indicate how cognition was measured as each study uses a different measurement and it would be important to indicate how they differ.

***Our response:*** *The discussion section has been modified to clarify this distinction.*

As the outcomes were focused on dietary, there was sparse information in the discussion that focused on these results and why.

***Our response:*** *As per our research, our outcome variable is Cognitive function, not dietary intake. Therefore, we discussed this. Also, we have discussed dietary intake.*

**Reviewer #2:** It is an existing knowledge that poor nutrition and dietary intake interfere with cognitive development in children. Though the study is the first ever study (according to author declaration), it was better to draw some novel inference from the findings and discuss accordingly.

This is an interesting study on the association between nutritional status, dietary habits and cognitive function among children in Bangladesh. Nutritional status and dietary habits is crucial for cognitive development of children.

I propose to address the following issues.

**Abstract:**

Line 16: May the research is not limited worldwide. The author needs to focus on Bangladesh and need to give background about Bangladesh.

***Our response:*** *The introduction section has been modified to contexualize the situation in Bangladesh.*

**Introduction:**

Line 49: The author stressed on the need of only micronutrients, however, macronutrients like protein and fat is also important for the development of nervous system.

***Our response:*** *Although macronutrients are also important for the development of the nervous system, the focus of this study was to explore whether there is any association between micronutrients and cognitive functions.*

Line 72: To develop proper rationale of the study, author need to provide some background or previous studies in the related field done in Bangladesh (if have).

***Our response:*** *To date, there is no study in Bangladesh that looked at cognitive functions and their relation to micronutrients among school children. However, a few related studies have been incorporated to rationalize this study as suggested.*

**Methods:**

Line 81: Why did you choose children between 8-12 years, however, in the title it has been mentioned that the study was done in school going children. Age range of school going children could 5 to 15 Years. As the age range 8-14 years, is this representative to the schooling children?

***Our response:*** *The study included participants from both private and Government schools. In private schools, the children usually complete two years of pre-schooling before entering grade 1, which is quite different from that of the government schools. Although the government school age starts at 6 years in Bangladesh, for private schools, the age is 8 years. This is why the age range 8-14 was considered for robust comparison. We stated this in the title of the revised manuscript as well.*

Line 106: Is the PedsQL and FFQ validated in Bangladesh? How the author ensured the quality of FFQ as these needs to depends on the memory of children for their food intake.

***Our response:*** *Yes, both the scales are validated in Bangladesh (references in method section)****.*** *References for validation of PedsQL and FFQ have been included. The FFQ was self-reported by the parents. Also, a helpline number was included to answer any queries that may arise while completing the questionnaire.*

Line 119: The does not need to include questions in this section. The author can upload questions file separately as supplementary file.

***Our response:*** *The questions of the pedsQL scale have been deleted from the manuscript as per your suggestion. PedsQL is a brief questionnaire and is readily available on the web.*

Line 150: The author conducted few simple statistical analyses like regression and descriptive analysis. It might be better to perform few advanced statistical analyses like logistic regression.

***Our response:*** *Thank you for the suggestion. This study is primarily focused on understanding any association between nutritional status, dietary habits, and cognitive function of school aged children in Bangladesh. The findings of this study mostly indicate further exploration through longitudinal studies and clinical trials. However, the unavailability of multivariate analysis has been mentioned in the limitations.*

**Results:**

Line 176: In Table 2 author need to provide the BMI for age Z score cut-off points for BMI categories.

***Our response:*** *The* *BMI for age Z score cut-off points for BMI categories have been added in Table 2 as suggested.*

**Discussion:**

It is an existing knowledge that poor nutrition and dietary intake interfere with cognitive development in children. Though the study is the first ever study (according to author declaration), it was better to draw some novel inference from the findings and discuss accordingly.

***Our response:*** *The discussion section has been modified accordingly to the reviewer’s suggestion.*

**References**

1.	Perneger T V., Courvoisier DS, Hudelson PM, Gayet-Ageron A. Sample size for pre-tests of questionnaires. Qual Life Res [Internet]. 2015 Jan 1 [cited 2023 Dec 17];24(1):147–51. Available from: https://pubmed.ncbi.nlm.nih.gov/25008261/
